# A ten-year overview of cancer genetic family history screening in Georgia’s Latina population

**DOI:** 10.3389/fpubh.2024.1432971

**Published:** 2024-10-02

**Authors:** D. Ramirez Leon, L. E. Barber, S. Gabram-Mendola, C. Snyder, S. T. Vadaparampil, L. Fuzzell, L. E. McCullough, L. Durham, Y. Guan

**Affiliations:** ^1^Department of Behavioral, Social, and Health Education Sciences, Rollins School of Public Health, Emory University, Atlanta, GA, United States; ^2^Department of Epidemiology, Rollins School of Public Health, Emory University, Atlanta, GA, United States; ^3^Georgia Center for Oncology Research and Education, Atlanta, GA, United States; ^4^H. Lee Moffitt Cancer Center and Research Institute, Tampa, FL, United States

**Keywords:** Hispanic/Latina, hereditary breast cancer, family history and cancer, genetic screening, rural health, area deprivation index (ADI), rural urban comparison

## Abstract

**Background:**

Population-based cancer genetic family history (FH) screening to identify families at high risk for BRCA-associated cancers has been endorsed by national public health policies. This report aimed to describe the utilization of FH screening services from 2013 to 2022 according to rurality and socioeconomic deprivation among Latinas in Georgia.

**Methods:**

Women who attended a medical appointment at participating Georgia Public Health Clinics were invited to complete FH screening. Screening results and participant zip code were reviewed. Area deprivation index (ADI) was measured at the census block group level and dichotomized (more deprived and less deprived). Rurality was measured through Rural–Urban Commuting Area (RUCA) codes and dichotomized (urban and rural). The ADI and RUCA codes were linked to participant data by zip code to characterize FH utilization among the Latina community.

**Results:**

Of the 9,330 adult Latinas in Georgia that completed cancer genetic FH screening, 9,066 (97.17%) women screened negative, and 264 (2.83%) screened positive (i.e., FH suggestive of higher risk for carrying BRCA1/2 mutations compared to the general population). Screening completion was higher among Latinas in urban areas (*n* = 7,871) compared to rural areas (*n* = 1,459). Screening completion was also higher in more socially deprived areas (*n* = 5,207) compared to less socially deprived areas (*n* = 4,123).

**Conclusion:**

Georgia’s FH screening program reached Latinas across Georgia, particularly those living in urban, socially deprived areas. To ensure equitable cancer genetic screening dissemination, future efforts should prioritize tailored outreach in rural regions and comprehensive evaluations to identify key determinants of screening trends among Georgia’s Latina population.

## Introduction

Breast cancer continues to be the most prevalent cancer among women, impacting 1 in 8 women throughout their lifetime (1). Of the over 310,000 new annual breast cancer cases diagnosed in the United States, it is estimated that 5–10% are due to hereditary gene mutations (2, 3). Brief family history assessments endorsed by national (e.g., United States Preventative Services Task Force [USPSTF]) and public health organizations (e.g., Centers for Disease Control and Prevention) now enable low-cost population-based screening to identify families at high risk for *BRCA*-associated cancers (4–6). Implementing this screening is critical as women who carry a *BRCA1/2* mutation have significantly increased lifetime risks for breast (50–80%) and ovarian (10–40%) cancer (7, 8). Life-saving prevention and treatment options are available to mutation carriers (7, 8). Unfortunately, current efforts to expand cancer genetic screening beyond urban cancer specialty settings that serve predominantly non-Hispanic White populations have been exceedingly slow and will likely lead to further entrenchment of health disparities (9–12).

Cancer genetic risk screening has notable benefits particularly relevant for Latino and Hispanic people (hereafter referred to as “Latinos”). Compared to non-Hispanic White women, Latinas are often diagnosed with breast cancer at younger ages and with tumor types (e.g., triple-negative disease) linked to hereditary genetic mutations (13–16). However, Spanish-speaking Latinas are half as likely as White individuals to have discussed genetic counseling or testing with a health care provider (11, 17).

Various factors may contribute to the low uptake of cancer genetic services among Latino communities, such as cost, language barriers, inadequate insurance coverage, lack of awareness on the part of the patient and/or provider, and limited availability of screening services (18). However, prior studies examining disparities in access have limited consideration of social determinants of health, such as neighborhood disadvantage and neighborhood access. To address disparities in cancer detection and outcomes, it is essential to understand health resource utilization using geographic and area-based metrics. For example, disparities in delayed breast cancer diagnosis and survival have been reported among racial/ethnic minorities with low socioeconomic status, census tract-level poverty, and inadequate health insurance (18–20). While cancer genetic risk screening options have become more widely available, uptake of services can vary based on rurality and geospatial disparities (21, 22). Although neighborhood disadvantage and access to health resources have been studied, the effects of both factors have not been considered in the completion of family history screening for breast cancer. For public health interventions targeting improved cancer genetic screening rates and reduced cancer-related mortality in the Latino community, it is essential to identify areas where individuals face the most significant challenges in accessing and using care. This paper seeks to address the limitations in prior research by considering the role of neighborhood disadvantage and neighborhood access (determined by area rurality) in cancer genetic screening completion.

Public health settings serving medically underserved, as well as rural, communities present ideal platforms to mitigate disparities in accessing cancer genetic services. Notably, since 2012, the state of Georgia has been a trailblazer in the implementation of a statewide program for family history-based screening for *BRCA*-associated cancers (23). The Women’s Health section of the Georgia Department of Public Health (DPH) supports and oversees the Georgia Center for Oncology Research and Education (GA CORE)‘s family history screening program through public health clinics across the state. The program has facilitated over 30,000 Georgian women in completing their family history assessments for BRCA-associated cancers. A significant portion of these women were uninsured and lived in poverty (23). It’s noteworthy that Latinos constitute 10% of Georgia’s population, making them the third-largest racial and ethnic group (23). Distressingly, a significant portion of the Latino community in Georgia faces financial challenges, with 21% living in poverty and 28% in low-income conditions. This group also registers the highest uninsured rate at 33% (24). While the program’s success in screening women is commendable, its efficacy in reaching the Latina community in Georgia remains underexplored.

The aim of this observational cross-sectional study was to describe the utilization of cancer genetic family history screening services from 2013 to 2022 among the Latina community in the state of Georgia, according to rurality and area-level socioeconomic deprivation.

## Methods

### Study population

Between January 2013 and June 2022 Georgia women aged ≥18 years with scheduled Women’s Health visits at one of the 81 participating Public Health Clinics in Georgia were approached by a nurse trained at the beginning of the program to complete the genetic risk assessment using the Breast Cancer Genetics Referral Screening Tool (B-RST^™^) (prior to 2021) or the Ontario Family History Assessment Tool (FHAT) (located on GA CORE’s website, www.georgiacancerinfo.org/breast-cancer-gene-screen/) (5, 23). Both tools are endorsed by the USPSTF as validated screening tools for identifying women who should be referred for genetic counseling and the FHAT is available in English and Spanish (5). Screening results indicate two categories of risk for BRCA-associated cancers: “positive” (high risk for BRCA1/2 mutation – genetic counseling recommended) and “negative” (unlikely to carry a BRCA1/2 mutation – genetic counseling not recommended). Patients who received a positive screening result were referred to the GA CORE advanced practice nurse in genetics (APNG) for further assessment. If found eligible for testing, the APNG coordinated saliva or blood collection and communicated test results to both the patient and the referring providers.

Between January 2013 – June 2022, more than 30,000 women have completed the family history screener (23) with 9,328 women self-identifying as Latino/Hispanic.

### Data collection

#### Family history screening completion

For each woman who completed the family history screening assessment between 2013–2022, Georgia CORE collected data on participant age, postal code, public health district of residence at the time of screening, as well as screening result (i.e., positive, negative).

#### Rurality

We defined urban and rural conditions using the 2010 Rural–Urban Commuting Area (RUCA) codes measured at the zip code level (25–28). RUCA codes classify census districts into one of four categories (urban, large rural city, small rural town, isolated small rural town) based on population density, urbanization, and daily commuting patterns (27). We aggregated 3 rural categories to 1 rural category due to limited sampling in the small rural and isolated rural zip codes. We dichotomized the RUCA codes into urban areas [1.0, 1.1, 2.0, 2.1, 3.0, 4.1, 5.1, 7.1, 8.1,10.1] and rural areas [4.0, 4.2, 5.0, 5.2, 6.0, 6.1, 7.0, 7.2, 7.3, 7.4, 8.0, 8.2, 8.3, 8.4, 9.0, 9.1, 9.2, 10.0, 10.2, 10.3, 10.4, 10.5, 10.6], which is commonly done in health research (29). RUCA codes were linked to participant data using 5-digit zip codes.

#### Area deprivation

The 2019 area deprivation index (ADI), available from the Neighborhood Atlas, was used as measure of the social and economic stability of a neighborhood (30). The ADI was constructed using 2015–2019 American Community Survey data on 17 indicators representing poverty, housing, employment, and education for zip codes in the state of Georgia. Using principal component analysis, indicators were weighted to create an ADI score, which was categorized into deciles, such that 1 indicated the lowest and 10 indicated the highest deprivation. For this analysis, we dichotomized ADI so that values of 0–5 indicated less deprivation and values of 6–10 indicated more deprivation. ADI can be linked to participant-level data based on a 9-digit zip code. However, the Georgia CORE only ascertained participants’ 5-digit zip code. Thus, the first 5 digits of the 9-digit zip codes were used to link the ADI to participant data. The first 5 digits of the 9-digit zip code define a relatively broad geographic area, while the last 4 digits identify smaller units within the broad geographic area. Therefore, using 5-digit zip codes to link participant data to the ADI resulted in a one-to-many match. Using a similar approach as another study with the zip code limitation, we calculated the median ADI value for each 5-digit zip code and then categorize them dichotomously using a median split (31). If more than 50% of the ADI matches consisted of ADI values <6, participants were categorized as living in a less deprived area. If more than 50% of the matches consisted of ADI values ≥6, participants were categorized as living in a deprived area.

### Statistical analyses

Descriptive statistics were used to describe family history screening completion, rurality, and area deprivation. We plotted yearly trends in screening completion. Additionally, we conducted a sensitivity analysis to explore potential misclassification of area deprivation. In this sensitivity analysis, we recalculated participants’ ADI using a weighted average approach and redescribed family history screening completion according to the new ADI classification. Analyses were completed using SAS 9.2 (Cary, North Carolina).

## Results

### FH screening program reach

A total of 9,330 adult Latinas in Georgia completed FH screening from January 2013–June 2022. This number accounts for about 3.28% of the adult Latina population in Georgia, with the total number of adult Latinas calculated by summing the number of adult Latinas in every county in Georgia in the 2021 United States Census data (Table 1, total number of Latinas = 284,160) (32). The age at time of screening ranged from 18–85 years (median = 36.00 ± 9.83 years). Of the Latinas that completed FH screening, 9,066 (97.17%) received a negative result and 264 (2.83%) received a positive result.

**Table 1:** Total Latinas screened in each public health district, assessed by ADI and RUCA.

GA public health districts	Total # of adult Latinas in PHD	Total # of Latinas screened	ADI		RUCA	
			Less deprived	More deprived	Urban	Rural
3–4 Gwinnett, Newton & Rockdale	70,914^a^	202 (2.17%)	143 (70.79%)	59 (29.21%)	202 (100%)	0 (0%)
3–1 Cobb–Douglas	38,566	233 (2.50%)	220 (94.42%)	13 (5.58%)	231 (99.14%)	2 (0.86%)
2 North	26,743^a^	1,053 (11.29%)	377 (35.80%)	676 (64.20%)	894 (84.90%)	159 (15.10%)
3–2 Fulton	26,134	17 (0.18)	13 (76.47%)	4 (23.53%)	17 (100%)	0 (0%)
1–2 North Georgia	22,760^a^	15 (0.16)	4 (26.67%)	11 (73.33%)	15 (100%)	0 (0%)
3–5 DeKalb	19,275^a^	31 (0.33%)	15 (48.39%)	16 (51.61%)	31 (100%)	0 (0%)
4 District 4	16,144^a^	1,337 (14.33%)	431 (32.24%)	906 (67.76%)	1,288 (96.34%)	49 (3.66%)
3–3 Clayton	11,805	2 (0.02%)	0 (0%)	2 (100%)	2 (100%)	0 (0%)
1–1 Northwest	11,464^a^	598 (6.41%)	117 (19.57%)	481 (80.43%)	532 (88.96%)	66 (11.04%)
9–1 Coastal	9,856^a^	21 (0.23%)	6 (28.57%)	15 (71.43%)	21 (100%)	0 (0%)
10 Northeast	8,791^a^	182 (1.95%)	117 (64.29%)	65 (35.71%)	151 (82.97%)	31 (17.03%)
6 East Central	7,698^a^	75 (0.80%)	49 (65.33%)	26 (34.67%)	73 (97.33%)	2 (2.67%)
5–2 North Central	6,047^a^	42 (0.45%)	17 (40.48%)	25 (59.52%)	41 (97.33%)	1 (2.38%)
7 West Central	5,686^a^	35 (0.38%)	4 (11.43%)	31 (88.57%)	33 (94.29%)	2 (5.71%)
8–1 South	2,277^a^	672 (7.20%)	21 (3.12%)	651 (96.88%)	374 (55.65%)	298 (44.32%)
5–1 South Central	0^a^	–	–	–	–	–
8–2 Southwest	0^a^	718 (7.70%)	9 (1.25%)	709 (98.75%)	120 (16.71%)	598 (83.29%)
9–2 Southeast	0^a^	–	–	–	–	–
Unknown PHD	–	4,097 (43.91%)	2,580 (62.97%)	1,517 (37.03%)	3,846 (93.87%)	251 (6.13%)
Total	284,160	9,330 (100%)	4,123 (44.19%)	5,207 (55.81%)	7,871 (84.36%)	1,459 (15.64%)

a Indicates the 2021 US Census reported missing data from at least 1 county in the corresponding public health district.

ADI, Area Deprivation Index; PHD, Public Health District(s); RUCA, Rural–Urban Commuting Area.

In total, 107 of the 264 (40.53%) eligible Latinas opted to receive genetic counseling and 103 Latinas (96.36%) completed genetic testing. Of the 103 Latinas who completed hereditary cancer screening multigene panel testing, 57 (55.34%) women received a negative result (i.e., no pathogenic variants were found), 37 (35.92%) women received a negative result with a second result of a variant of uncertain significance (VUS), 4 (3.88%) women received a positive result (i.e., a pathogenic variant was found), and 5 women received a positive result with a second result of a VUS (4.85%).

**Figure 1:**
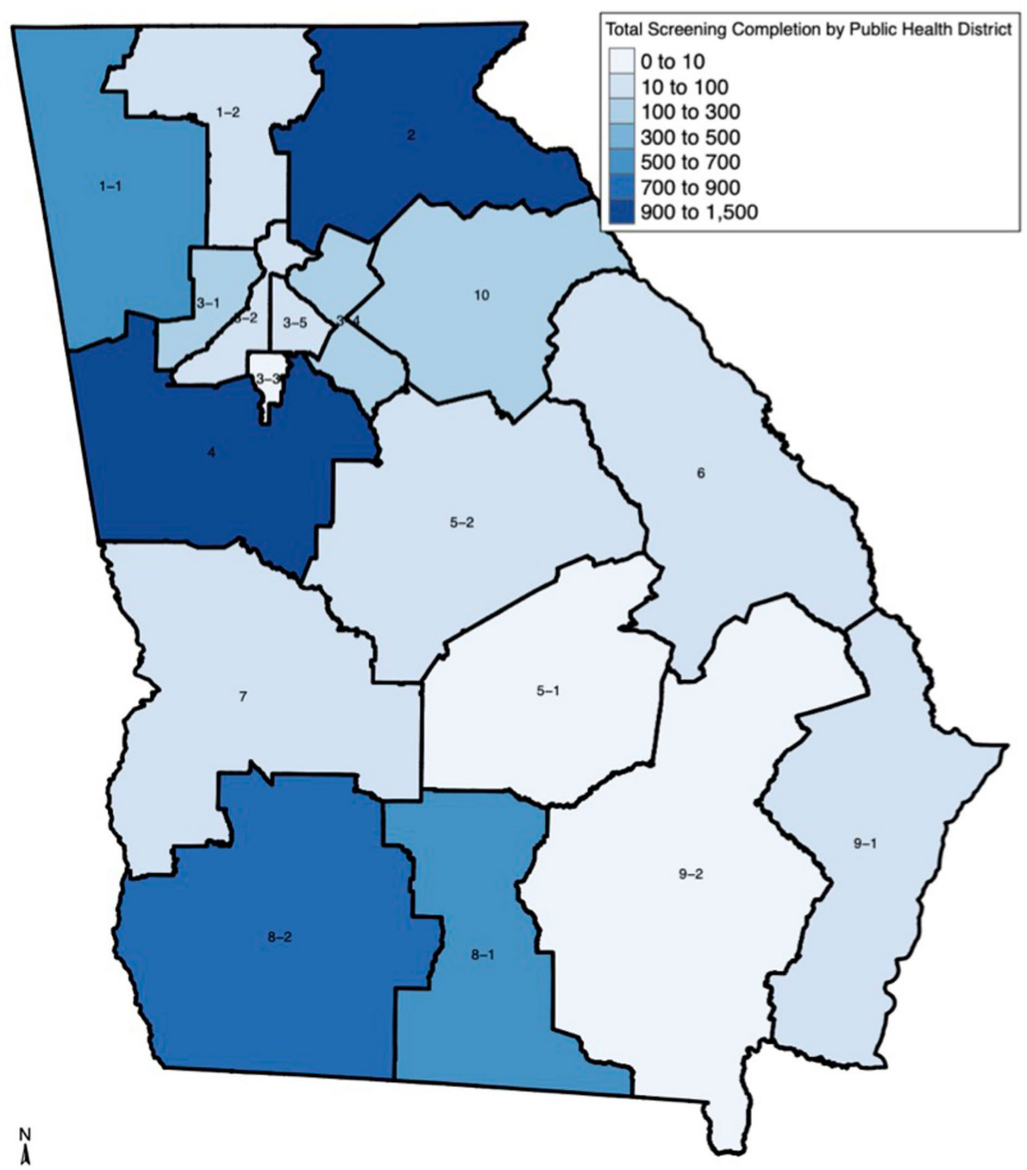
Family history screening completion by Georgia Public Health District. The map displays the following public health districts: 1–1 Northwest (Rome); 1–2 North Georgia (Dalton); 2 North (Gainesville); 3–1 Cobb–Douglas; 3–2 Fulton; 3–3 Clayton (Jonesboro); 3–4 GNR (Lawrenceville); 3–5 DeKalb; 4 District 4; 5–1 South Central (Dublin); 5–2 North Central (Macon); 6 East Central (Augusta); 7 West Central (Columbus); 8–1 South (Valdosta); 8–2 Southwest (Albany); 9–1 Coastal (Savannah); 9–2 Southeast (Waycross); 10 Northeast (Athens).

FH screening was completed by Latinas in 16 of 18 (88.89%) state public health districts (PHD). Table 1 and Figure 1 show FH screening completion varied greatly across the 16 public health districts (mean = 327 women, range: 2–1,337). The four public health districts with the highest proportion of FH screening were District 4 (*n* = 1,337, 14.33%), District 2 North (*n* = 1,053, 11.29%), District 8–2 Southwest (*n* = 718, 7.70%), and District 8–1 South (*n* = 672, 7.20%), while District 3–3 Clayton had the lowest (*n* = 2, 0.02%). Most of the participating public health clinics were in urban (*n* = 106, 60.23%) compared to rural (*n* = 70, 39.77%) areas and in more deprived (*n* = 140, 79.55%) compared to less deprived areas (*n* = 36, 20.45%).

### FH screening completion by rurality and area deprivation level

A greater proportion of Latinas who completed the FH screening resided in urban areas (*n* = 7,871, 84.36%), as well as regions characterized by higher deprivation (*n* = 5,207, 55.81%). Conversely, a smaller segment lived in rural areas (*n* = 1,459, 15.64%) and less deprived regions (*n* = 4,123, 44.19%).

Notably, 5 (27.78%) Public Health Districts (3–4 Gwinnett, Newton & Rockdale, 3–1 Cobb Douglass, 3–2 Fulton, 10 Northeast, and 6 East Central) reported a greater FH screening among women from less deprived areas compared to the other participating PHDs. Of all participating PHDs, 8–2 Southwest was the only district that reported higher FH completion in rural areas (*n* = 598, 83.29%) compared to urban areas (*n* = 120, 16.71%).

### FH screening completion over the years

The number of Latinas completing FH screening increased from 377 in 2013 to 1,787 in 2016. However, since 2016, overall FH screening completion has decreased to an average of 998 women screened per year.

**Figure 2:**
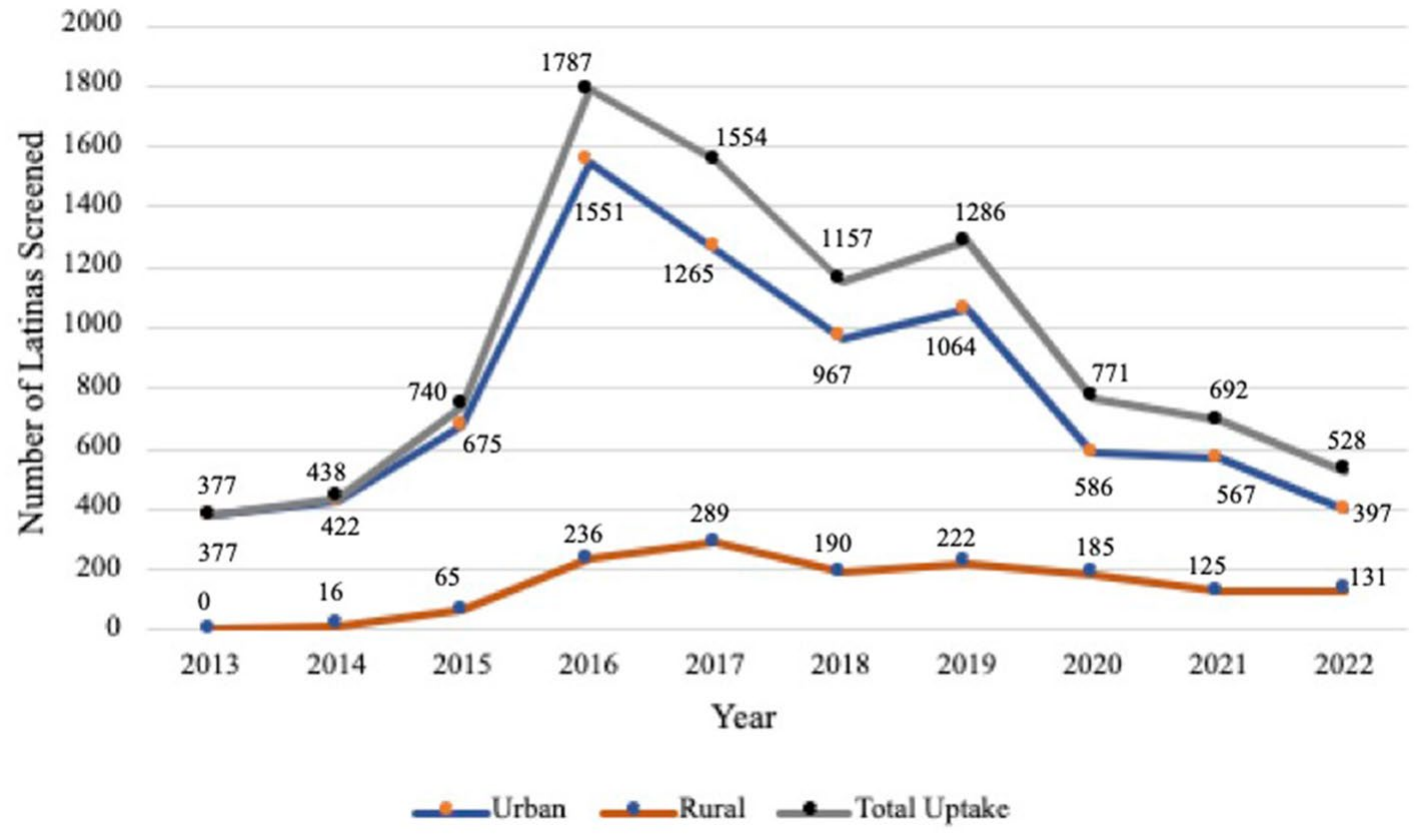
Family history screening completion assessed by rural–urban commuting area (RUCA) codes and year of completion.

FH screening completion has been consistently higher in urban areas compared to rural areas (Figure 2), with the largest difference recorded in 2016 (urban *n* = 1,551, rural *n* = 236, difference = 1,315). However, since 2020, the urban–rural gap in FH screening completion has narrowed significantly, with the smallest difference recorded in 2022 (urban *n* = 397, rural *n* = 131, difference = 266). Figure 2 displays that the narrowing between categories is due to reduction in testing across the state.

**Figure 3:**
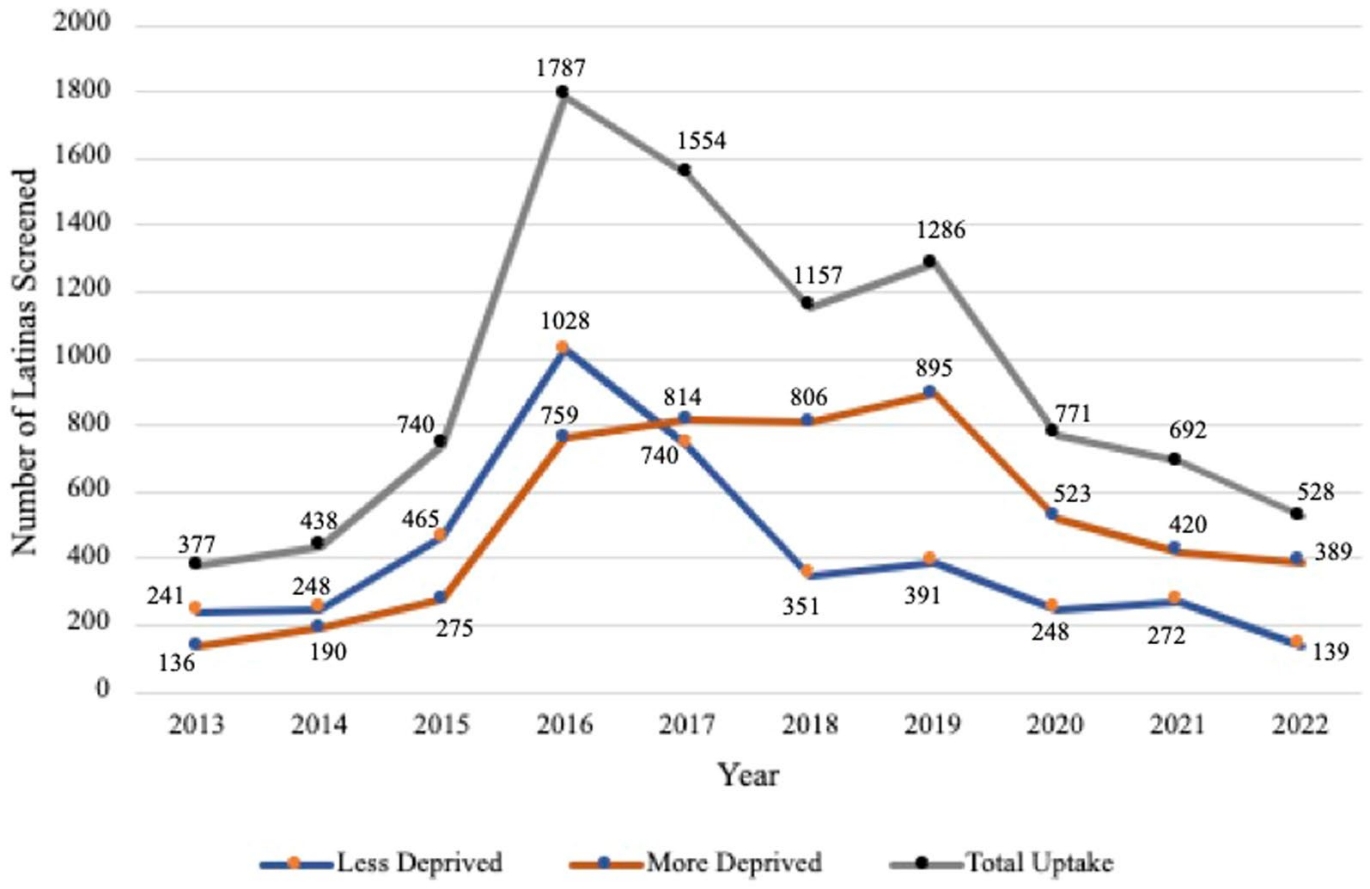
Family history screening completion assessed by area deprivation index (ADI) scores and year of completion.

FH screening completion among Latinas was higher in less deprived areas from 2013–2016 (Figure 2). However, after 2016, FH screening completion was consistently higher in more deprived areas, with the largest gap between categories recorded in 2019 (more deprived = 895, less deprived = 391, difference = 504). Since 2020, the gap has narrowed significantly with the smallest difference observed in 2021 (more deprived = 420, less deprived = 272, difference = 148) (see Figure 3).

## Discussion

Georgia’s FH screening program was able to reach Latinas across the state of Georgia, particularly those living in urban, socially deprived areas. Our systematic review suggests that implementing family history-based screening in public health settings (e.g., local healthcare call centers, community healthcare practices) that already reach well-delineated target populations has successfully increased the uptake of cancer genetic counseling, especially for ethnic minorities and those living in low-resource settings (33). Similarly, Latinas who live in more deprived areas are more likely uninsured and live in poverty, thus more likely to rely on services provided by Public Health Clinics. Additionally, rural residents, potentially hindered by transportation issues, have not been able to fully utilize the program as screenings are currently exclusively offered in person. However, overall participation in the FH screening program and uptake of genetic testing is low, and its implementation varies across public health districts. Furthermore, the FH screening program is currently only offered in the women’s health section, thus reducing early detection and prevention opportunities for hereditary cancers in both men and their families. Although 264 Latinas were eligible to receive no-cost or low-cost genetic counseling and testing, only 40.53% opted to receive these services. The low uptake of these services may be attributed to language barriers, low genetics knowledge and numeracy, language and communication barriers between patients and healthcare providers, or concerns regarding health insurance coverage (34). To maximize the efficiency of this statewide screening framework, we need targeted outreach strategies, especially for rural Latina populations.

Between 2013 and 2016, there was a noticeable increase in the number of Latinas undergoing FH screening, but this number began to decline after 2016. Data indicates that urban areas consistently had higher screening completion rates compared to rural areas, but this disparity has been decreasing since 2020. Interestingly, while screenings in less deprived areas surpassed those in more deprived ones from 2013–2016, this dynamic shifted after 2016. Several factors might have influenced these trends. Beginning in 2013, educational outreach to health care providers expanded from one PHD to multiple districts, potentially contributing to the initial increase in screenings. The widespread public awareness increased by Angelina Jolie’s op-ed regarding genetic testing for hereditary cancers in 2013 might also have played a role. On the other hand, the decline in screenings after 2016 could be attributed to staffing changes, limited Spanish-speaking healthcare staff available, and shifts in clinic priorities potentially influenced by localized social events, such as an increase in neighborhood violence leading to clinic shutdowns. However, the exact reasons behind these shifts among the Latina community remain unknown. It is crucial to conduct a systematic evaluation to gain a comprehensive understanding into these changes.

This brief research report comes with some limitations that should be considered when interpreting the study findings. The FH screening program only records self-reported 5-digit zip codes, which may have resulted in misclassification of area deprivation status. Moreover, there was inconsistent documentation regarding whether zip code represented the patient’s address or the clinic’s address across different clinic sites. Consequently, our method of determining participants’ ADI and rurality may not be entirely accurate. However, results were similar in our sensitivity analysis in which ADI was calculated using a weighted average approach and then dichotomized into less and more deprived area, suggesting less potential for misclassification. Furthermore, our calculation of screening uptake rates relied on population-based census data as the denominator rather than the total number of Latina women invited to participate in the screening program. This limited our capacity to analyze statistical variations in screening rates by ADI and RUCA categories. Future efforts should emphasize refining data collection and documentation procedures.

## Conclusion

This study found that the state hereditary cancer screening program was able to reach Latinas across the state of Georgia, particularly those living in urban, socially deprived areas. This state initiative is especially crucial for Latinas without health insurance coverage who rely on accessible public health services. It is important to continue assessing the determinants that affect screening patterns among the Latina population in Georgia’s urban and rural areas. Such evaluations will enable the development of tailored outreach strategies to achieve equitable cancer genetic screening dissemination.

The results of the study will guide a thorough assessment involving public health services throughout Georgia, offering insights into organizational capabilities, and identifying barriers and facilitators to program implementation. By leveraging existing public health infrastructure, our program offers a potential sustainable outreach strategy to increase the reach of cancer genetic services, thereby expanding the accessibility of cancer genetic services to a more diverse audience.

## Data Availability

The raw data supporting the conclusions of this article will be made available by the authors, without undue reservation.
